# Comment on Roman-Filip et al. Non-Aneurysmal Perimesencephalic Subarachnoid Hemorrhage: A Literature Review. *Diagnostics* 2023, *13*, 1195

**DOI:** 10.3390/diagnostics13223463

**Published:** 2023-11-17

**Authors:** Ajay Malhotra

**Affiliations:** Department of Radiology and Biomedical Imaging, Yale School of Medicine, New Haven, CT 06501, USA; ajay.malhotra@yale.edu

We would like to congratulate Roman-Filip et al. on their recent review on perimesencephalic hemorrhages [1]. We have a few comments about the diagnostic approach to perimesencephalic hemorrhages.

Noncontrast CT has been shown to have very high sensitivity and negative predictive value for subarachnoid hemorrhages in the first 6 h of the onset of a thunderclap headache [2]. Although CTA is being increasingly used by emergency physicians due to the ease of ordering and the shorter associated ED stay, it is important to keep in mind that CTA is detecting aneurysms and not intracranial bleeds [3,4]. Lumbar punctures may be more appropriate for the detection of subarachnoid hemorrhages [5]. The detection of incidental aneurysms leads to a dilemma, and CTA is not cost-effective in looking for subarachnoid hemorrhages in patients with thunderclap headaches [6,7,8].

A small proportion of perimesencephalic hemorrhages are caused by posterior circulation aneurysms. However, there is limited utility for any further imaging after a negative CTA in patients with perimesencephalic hemorrhages [9]. Further imaging may not be cost-effective given its low-utility [10,11,12]. However, the use of repeat imaging remains prevalent, and the previous literature seems to have had limited impact on actual clinical practice [13].

Recurrence is rare, and prognosis in perimesencephalic hemorrhages is relatively better compared to aneurysmal subarachnoid hemorrhages [14].
